# *In vitro* and *in vivo* characterization of a novel HSV-1 H129 neurotracer recombinant reveals dose-dependent replication and spread dynamics

**DOI:** 10.1128/jvi.02021-25

**Published:** 2026-01-08

**Authors:** Taulima Nua, Eric Velazquez-Rivera, Ilayda Sayin, Jessica C. Ogu, Michele Wu, Xiangmin Xu, Orkide O. Koyuncu

**Affiliations:** 1 Department of Microbiology and Molecular Genetics, School of Medicine, University of California 8788, Irvine, California, USA; 2 Department Anatomy & Neurobiology, School of Medicine, University of California 8788, Irvine, California, USA; 3 Center for Neural Circuit Mapping, School of Medicine, University of California 8788, Irvine, California, USA; Dartmouth College Geisel School of Medicine, Hanover, New Hampshire, USA

**Keywords:** viral neurotracer, anterograde spread, H129, replication defective HSV-1

## Abstract

Understanding the organization and connectivity of brain circuits has been a major focus of neuroscience for decades, and viral vectors have been commonly utilized to map neural connections in animal models. The alpha-herpesvirus herpes simplex virus 1 (HSV-1) is one of the most studied and genetically modified viruses due to its bidirectional spread capabilities in the nervous system and replication dependency on the viral thymidine kinase (TK) gene in the terminally differentiated neurons. H129, a brain isolate of HSV-1, is the most promising anterograde neurotracer that has been extensively used to map neuronal projections. Replication-deficient monosynaptic H129 vectors (H129-dTK) have been previously developed. In this study, we report the development and characterization of a new H129-dTK recombinant that expresses mNeonGreen along with FlpO recombinase. We investigated the replication and spread properties of this recombinant utilizing compartmentalized primary neuron culture and FlpO/Cre reporter mouse lines. Our findings reveal that the replication, labeling, and spread phenotypes of H129-dTK recombinants strongly depend on the infecting dose of virus. At higher viral doses, H129-dTK exhibits limited replication and anterograde transsynaptic spread without requiring exogenous TK complementation, which can be visualized using Cre/FlpO reporter mice. This controlled replication reduces tissue damage at the injection site while allowing precise labeling of neuronal output projections. Together, these results provide valuable insights into the replication and spread characteristics of replication-deficient H129 recombinants in neurons *in vitro* and *in vivo*, supporting further development of H129-based neurotracers.

**IMPORTANCE:**

Dissecting brain circuit connectivity and organization is crucial for advancing our understanding of neural function and behavior. The herpes simplex virus 1 strain H129 is an effective neurotracer due to its predominant anterograde spread, though its replication often leads to tissue damage. To address this limitation, H129 recombinants lacking the thymidine kinase (TK) gene were developed to reduce replication in the nervous system. However, the absence of TK also limits reporter gene expression and neuron labeling. In this study, we engineered three novel H129-dTK recombinants that conditionally or constitutively express mNeonGreen along with FlpO/Cre recombinases. These recombinants showed dose-dependent replication and spread capacities, enabling efficient transsynaptic labeling without TK complementation. Additionally, recombinase-mediated labeling enables circuit tracing even under attenuated replication, providing versatility in circuit mapping. Our findings represent a significant advancement in neural circuit tracing, offering high specificity, reduced tissue damage, and precise, reliable labeling of neuronal pathways.

## INTRODUCTION

The mammalian nervous system has a complex network of neural circuits. Understanding the organization and connectivity of these circuits has been a major focus of neuroscience for decades. Viral vectors have been utilized to map neural circuit connections in animal models. Among these viral tracers, the alpha-herpesvirus (*α*-HV) herpes simplex virus 1 (HSV-1) is one of the most studied and manipulated viruses. *α*-HVs are large double-stranded DNA viruses containing a complex tegument layer and a lipid envelope (1). They can encode 70–200 genes during a productive infection and yield several hundred progenies per infected cell (1–3). *α*-HVs primarily invade the peripheral nervous system to establish life-long latency, but infections can spread to the central nervous system by passing multiple synapses to spread to higher-order neurons (4–6). Interestingly, *α*-HVs can spread bidirectionally in the nervous system from a presynaptic neuron to a postsynaptic neuron (anterograde) or from a postsynaptic neuron to a presynaptic neuron (retrograde) (6–8). Because of these transsynaptic spread properties, *α*-HVs have been used to trace neuronal circuits *in vivo,* including the projections to and from the primary motor cortex (6), trigeminal pathways from tooth pulp to cortex (9), subcortical projections to the inferior parietal lobule (10), and the contribution of brainstem nuclei in sensory airway inputs (11).

HSV-1’s capacity to infect various animal models, including primates, rodents, and rabbits, has made it a valuable tool for mapping neuronal circuits (5, 12). This is achieved by harnessing the virus’s ability to spread through chains of synaptically connected neurons via a process known as transsynaptic spread. α-HVs are unique in their bidirectional spread, facilitated by retrograde and anterograde transport mechanisms that rely on distinct microtubule motors (8, 13). After viral replication and capsid assembly in the nucleus, the virion undergoes secondary envelopment in trans-Golgi-like membranes, resulting in mature virions contained within cytoplasmic vesicles, ready for neuronal spread either from a presynaptic to a postsynaptic neuron (anterograde) or from a postsynaptic neuron to a presynaptic neuron (retrograde) (14–17). Since anterograde spread requires a specialized axonal sorting machinery, many of the other neurotropic viruses (e.g., rabies virus) predominantly spread in a retrograde manner. Although there are a few examples of other viruses that can spread anterogradely, their sorting and spread mechanisms are not well understood. For α-HVs, the anterograde spread machinery involves three viral envelope proteins (gE/gI/Us9) and their interaction with neuronal kinesins (15, 16).

The HSV-1 patient isolate, H129, was recovered from the brain of a patient suffering from viral encephalitis (6, 18). H129 shows a dominant anterograde sorting phenotype in the CNS, although the mechanism of this sorting bias has not been identified (6, 18, 19). Recombinant H129 neurotracer viruses expressing fluorophores predominantly label outputs of infected neurons, and hence they have been used for neural circuit tracers (5). Although the H129-based recombinant viruses are among the most promising anterograde tracers, there is a paradoxical challenge: H129 replication in the brain, while increasing the labeling intensity, leads to neuronal toxicity and death of the infected animals in a short period of time, thereby limiting the circuit analysis studies (20, 21).

To overcome this limitation, replication-deficient monosynaptic H129 recombinants were generated by conditionally expressing or deleting the viral thymidine kinase (TK) gene from the H129 genome (22, 23). TK synthesizes thymidine monophosphate by catalyzing the phosphorylation of thymidine, an essential step for the synthesis of that can be incorporated into the growing DNA chain by viral DNA polymerase (24). In proliferating cells, the endogenous TK compensates for viral TK deficiency to enable viral replication. However, cellular TK activity in non-proliferating cells, particularly in neurons, is very low (25). This strongly attenuates viral genome replication, thus preventing H129-dTK spread among connected neurons (11, 21, 22). TK-expressing helper adeno-associated virus (AAVs) have been used to complement replication of these viruses in the nervous system to restore viral replication, egress, and transsynaptic spread (26). Nevertheless, the restored replication leads to neuronal death in a short amount of time.

In this study, we addressed the toxicity associated with H129 replication-dependent gene expression and spread by examining dose-dependent replication and expression kinetics of dual reporter viruses. We generated three novel neurotracer recombinants: H129-dTK-mNeonGreen-P2A-FlpO (bright Flp; H129-BF), H129-dTK-mNeonGreen-P2A-Cre (H129-Cre), and H129-dTK-LSL-mNeonGreen-P2A-FlpO (dark Flp; H129-DF). When applied to transgenic reporter mouse models expressing Cre- or Flp-dependent fluorophores, these dual-reporter viruses enable neuronal labeling either directly (through replication-dependent fluorophore expression) or indirectly, via recombinase-mediated activation of endogenous reporters. mNeonGreen (mNG) is a strong fluorophore, but its detection requires abundant expression coupled to viral replication. By contrast, Cre and Flp recombinases can activate endogenous fluorophore expression in target cells even at low expression levels. Based on this, we hypothesized that the recombinase-mediated endogenous reporter expression could enable neuronal labeling even when H129 replication was attenuated by TK deletion and in the absence of a TK-expressing helper AAV.

Our results showed that H129-dTK recombinants are capable of replication in dissociated primary cortical neurons (CNs) without TK complementation, although with reduced virus yield. We characterized the infection and gene expression dynamics of H129-BF at both high and low multiplicity of infection (MOI), during retrograde (axon-to-cell body) and anterograde (cell body-to-axon) infection in a compartmented primary neuronal culture model. Retrograde infection efficiency of H129-BF was markedly reduced, particularly at low-dose axonal infections, while the anterograde infections showed dose-dependent replication and spread efficiency. We further tested the spread and labeling kinetics of H129-BF in two different reporter mouse models. When H129-BF replication was complemented by helper AAV vectors, both Flp reporter and mNG expression increased, leading to widespread infection and anterograde spread that caused tissue damage at the injection site within 5 days. Interestingly, the Flp-mediated endogenous reporter expression displayed distinct kinetics from virally encoded mNG, effectively decoupling neuronal labeling from viral replication efficiency. Finally, we found that H129-BF infections exhibit dose-dependent replication and spread in the mouse brain even without TK trans-complementation. At appropriate doses, this allowed efficient labeling of postsynaptic neurons in the contralateral site while avoiding significant tissue damage in the injection (ipsilateral) site.

## RESULTS

### Construction of new recombinants

**Fig 1:**
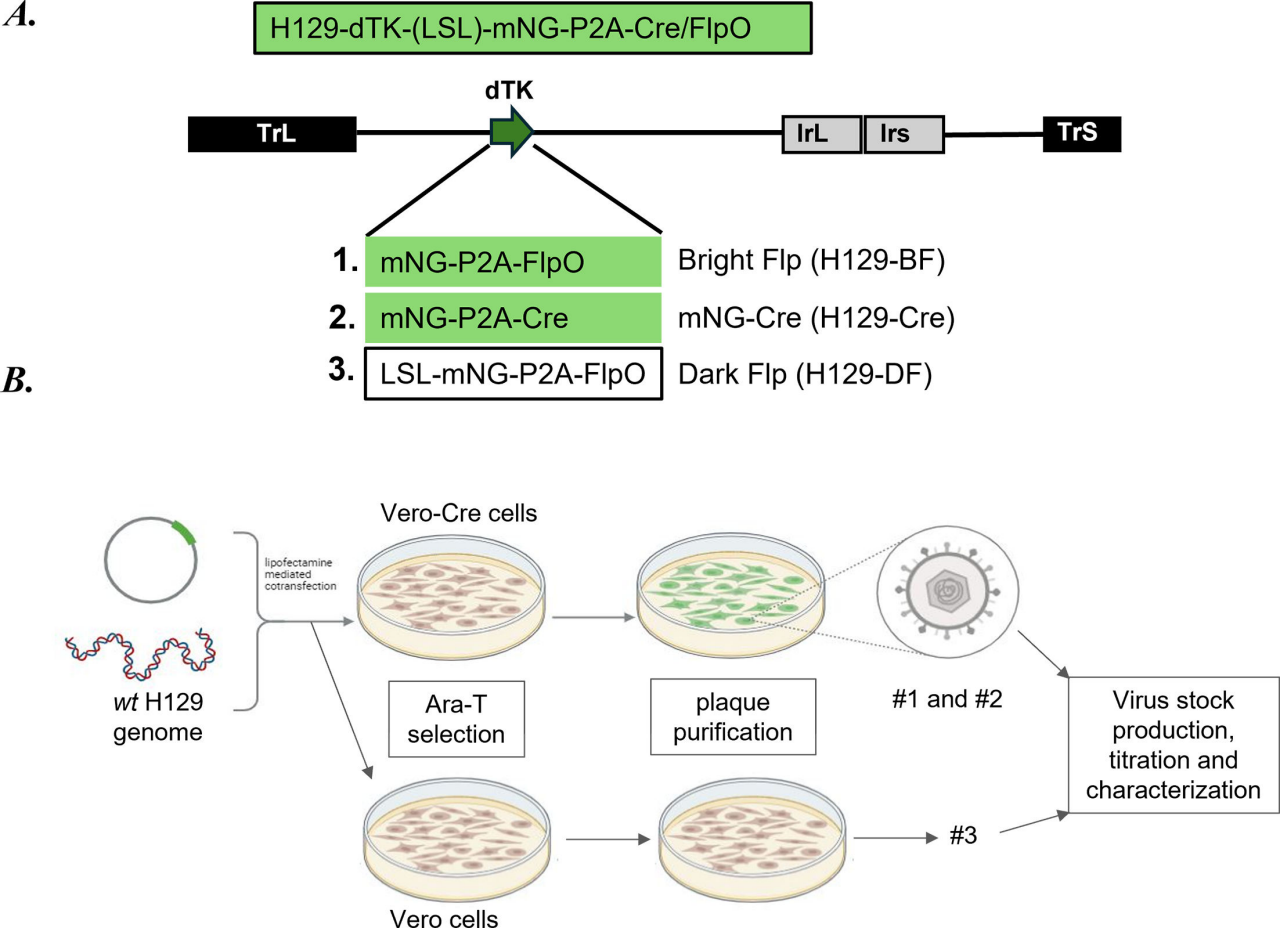
Construction of the new H129 recombinants. (**A**) H129 genome map showing the insert site (UL23) of mNG-P2A-recombinase (Cre or FlpO). (**B**) Simplified overview for homologous recombination and plaque purification of H129 recombinants using Vero-cre or Vero cells.

**Fig 2:**
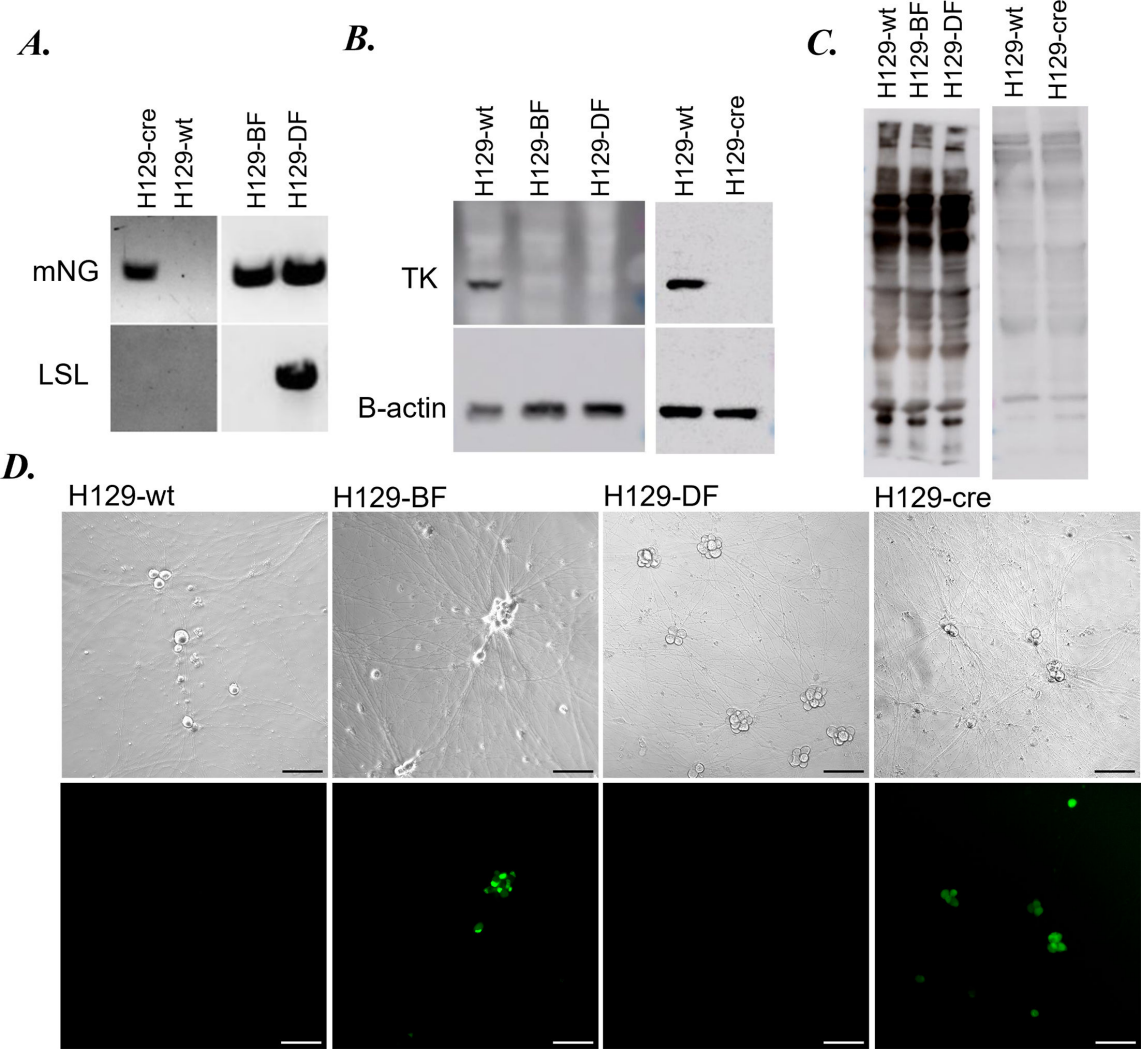
Verification of the novel H129 recombinants. (**A**) PCR amplification of the mNG (top) or lox-stop-lox region (bottom) using H129 recombinants or H129 wild-type virus as templates. (**B**) Western blots showing the presence or lack of HSV TK (top) expression. β-actin was used as a loading control (bottom). (**C**) Poly-HSV antibody was used to compare the structural protein expression levels of the recombinant viruses to wt H129. (**D**) Neurons were infected with the recombinant and wild-type H129 viruses to confirm mNG expression. Images were taken at 24 hpi. Scale bar: 100 µm.

We constructed three new replication-defective and conditional/constitutive recombinase-expressing H129 recombinants by simultaneously deleting the viral *TK* gene and replacing its coding sequence with (i) mNeonGreen-P2A-FlpO, (ii) mNeonGreen-P2A-Cre, or (iii) a Cre-dependent loxP-STOP-loxP(LSL)-mNeonGreen(mNG)-P2A-FlpO cassette (Fig. 1A) via homologous recombination in Vero or Vero-Cre cells (27, 28). H129 *wt* was purified and cotransfected with the linearized vector plasmid into host cells. H129 recombinants were isolated under Ara-T selection (Fig. 1B) and validated using PCR and Western blotting approaches (Fig. 2). The resulting H129 recombinants were named “H129-BF (bright Flp)” for (i) H129-dTK-mNG-P2A-FlpO; “H129-Cre” for (ii) H129-dTK-mNG-P2A-Cre; and “H129-DF (dark Flp)” for (iii) H129-dTK-LSL-mNG-P2A-FlpO. Viral TK protein expression was detected only in *wt* H129-infected cell lysate (Fig. 2B), while HSV-1 structural protein expression was comparable between H129 and all three recombinant virus-infected lysates (Fig. 2C).

**Fig 3:**
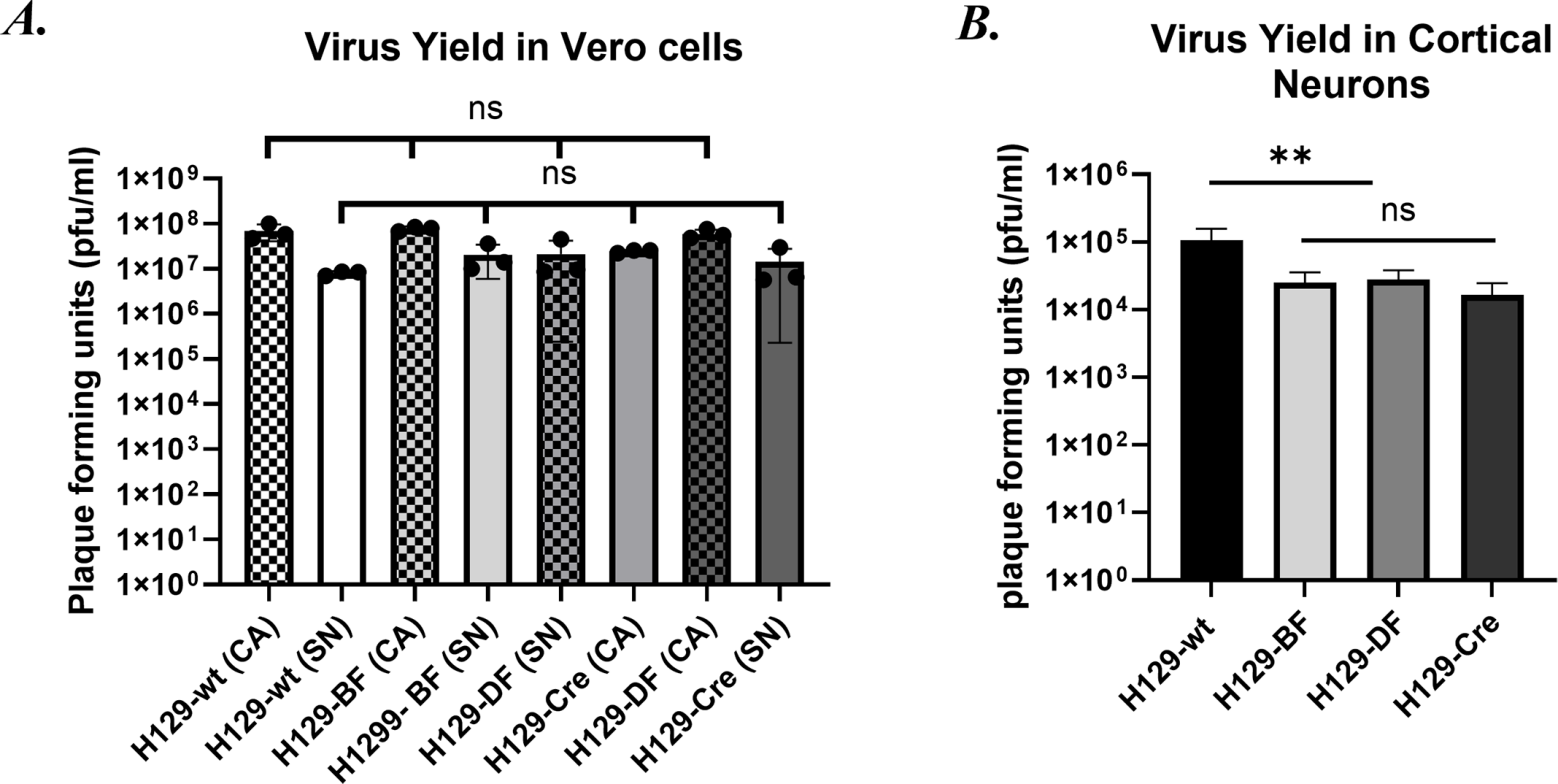
Replication efficiency of the new recombinants in Vero cells and primary CNs (*n* = 3). (**A**) Virus yields were determined in Vero cells infected at 1 MOI 24 hpi. SN = Supernatant; solid bars, CA = cell associated; checkered bars. (**B**) Virus yields in CNs at 1 MOI 48 hpi. Statistical significance was analyzed via Student’s t test, *P*-value of 0.004 is indicated with **, and *P*-values >0.05 are marked as not significant (ns).

Dissociated superior cervical ganglia (SCG) neurons were infected with *wt* H129 and the three new recombinants, and mNG expression was detected 24 hpi both in the cytoplasm and the nucleus of neuronal soma of H129-BF and H129-Cre-infected neurons (Fig. 2D). As expected, all three recombinants showed comparable cell-associated (CA) or supernatant (SN) virus yield to *wt* H129 at 24 h post-infection (24 hpi) in Vero cells infected at an MOI of 1 (Fig. 3A).

### Characterization of viral replication in primary CN culture

The replication defect of H129-dTK mutants in the nervous system has been shown in animal models, but their replication has not been well characterized in neuronal culture models. To compare the replication efficiency of the new H129-dTK recombinants to *wt* H129, we infected CNs isolated from E17 rat embryos at an MOI of 1 and determined the virus yield at 48 hpi (Fig. 3B). We observed mean reductions in viral titers of 7.3-, 3.3-, and 8.7-fold for H129-BF, H129-DF, and H129-Cre, respectively, when compared to *wt* H129, indicating that TK-null H129 recombinants retain replicative capacity in cultured CNs, albeit at a lower efficiency.

### Dose-dependent retrograde infection dynamics of H129-dTK-BF in compartmented SCG cultures

**Fig 4:**
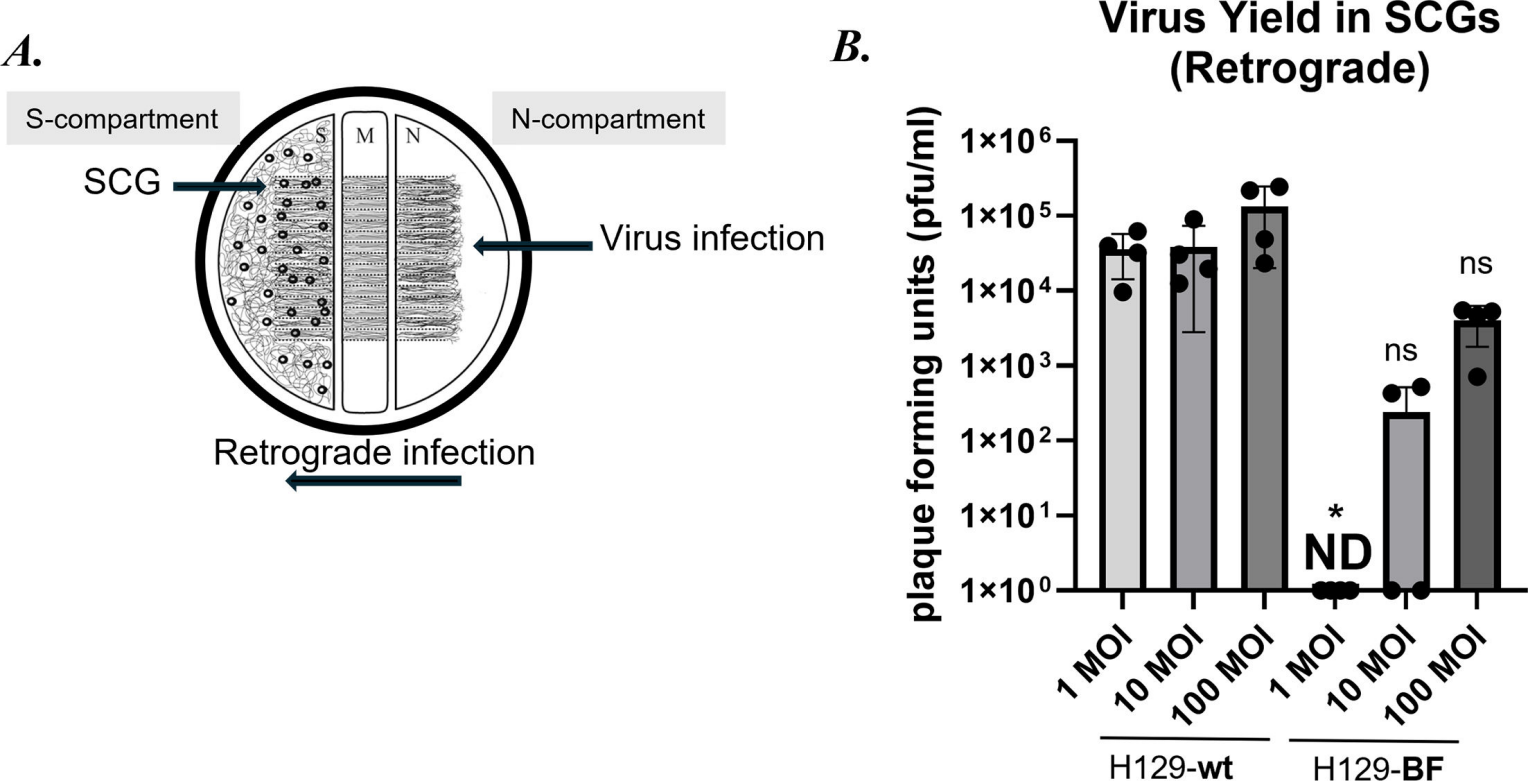
Retrograde infection efficiency of the new H129 recombinants in primary SCG neurons. (**A**) Diagram of the experimental setup. SCGs in tri-chambers were infected in the axonal (N-) compartment with wt and H129-BF viruses at 1, 10, and 100 MOIs. S-compartments were harvested at 72 hpi. (**B**) Virus yields were determined by titer assay on Vero cells. Statistical significance was analyzed via Student’s t test *P*-value of 0.016 is indicated with *, and *P*-values >0.05 are marked as not significant (ns).

To characterize the directional spread of H129-dTK mutants, we infected primary SCG neurons cultured in modified Campenot chambers consisting of three compartments (i.e., tri-chambers) (29, 30). For retrograde infection assays, neurons were infected at 1, 10, or 100 MOIs of *wt* H129 or the H129-BF recombinant in the axon-only N-compartments (Fig. 4A). After 72 h, viral yields reached 1.5 × 10^5^ (±5.6 × 10^4^ SEM) pfu/mL, 3.9 × 10^4^ (±1.7 × 10^4^ SEM) pfu/mL, and 3.6×10^4^ (±1 × 10^4^) pfu/mL in 100, 10, and 1 MOI *wt* H129 infections, respectively. In contrast, no virus was detected in the S-compartments of H129-BF-infected neurons at 1 MOI at 72 hpi. When the axonal compartments were infected with H129-BF at 10 MOI, we observed variable outcomes across biological replicates in the S-compartments, ranging from no detectable virus (ND) to very low titers, suggesting that the threshold for efficient replication is near 10 MOI, below which initiation of replication becomes inefficient. Overall, there was a significant reduction (695.7-fold mean decrease) in viral yield in the S-compartments of H129-BF compared to *wt* H129 at this MOI (Fig. 4B). At 100 MOI, H129-BF yielded 4 × 10^3^ (±1.1 × 10^3^) pfu/mL progeny representing an average 37.5-fold reduction compared to *wt* H129. These findings suggest that retrograde infection efficiency and/or replication is impaired in H129-TK-null recombinants relative to the *wt* H129 virus.

### Anterograde spread dynamics of H129-dTK-BF in compartmented SCG cultures

**Fig 5:**
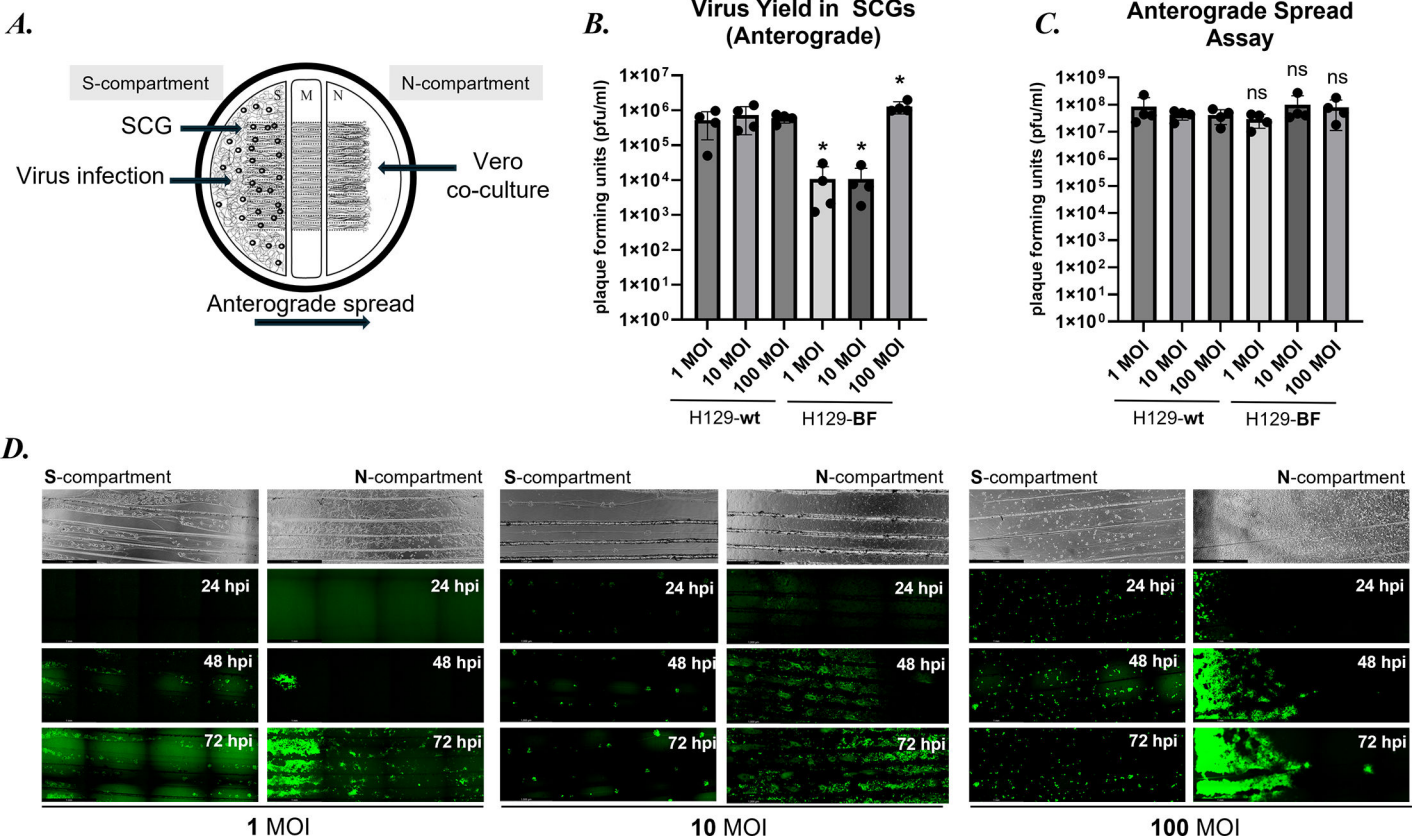
Anterograde transport and spread assay in primary SCG neurons. (**A**) Diagram of experimental setup. SCG cell bodies in the S-compartments were infected with the wt and H129-BF at 1, 10, and 100 MOIs. Vero cells were seeded on axons in the N-compartments. (**B**) S- and (**C**) N- compartments were harvested at 72 hpi, and virus yields were determined. (**D**) H129-BF-infected SCGs in the S-compartments and Vero cells in N-compartments were imaged at 24, 48, and 72 hpi. Scale bar: 1 mm. *P*-values of <0.05 are indicated with *, and *P*-values >0.05 are marked not significant.

To further characterize the spread dynamics of H129-dTK mutants, we performed anterograde spread assays in compartmented primary SCG cultures (Fig. 5A). The neurons were cultured in tri-chambers as described above. For anterograde spread assays, neurons were infected in the S- (soma and axons together) compartments at 1, 10, and 100 MOIs. Vero cells were seeded in the axon-only N-compartments to monitor anterograde sorting and spread efficiency of *wt* H129 and H129-BF. S- and N-compartments were harvested at 72 hpi for virus yield analysis. At 72 hpi, *wt* H129-infected neurons had significantly more virus yield in the S-compartments when compared to H129-BF-infected neurons, at 1 and 10 MOIs (48.8- and 68.7-fold mean difference, respectively) (Fig. 5B). Interestingly, at 100 MOI infections, H129-BF had approximately 2.1-fold more titer than *wt* H129, possibly due to the death of wt H129-infected neurons at this time point. The titers of both *wt* H129 and H129-BF-infected N compartments were comparable at 72 hpi (Fig. 5C). Since the TK-null H129 recombinants did not show any replication defect in Vero cells (Fig. 3A), we concluded that the replication and spread of *wt* and mutant H129 viruses among Vero cells reached a plateau at 72 hpi.

We also examined the replication and spread dynamics of H129-BF infection in both soma (S) and neurite (N) compartments by monitoring mNG accumulation over a time course of 24, 48, and 72 hpi at MOIs of 1, 10, and 100 (Fig. 5D). At 1 MOI, no detectable mNG signal was observed at 24 hpi, but fluorescence began to accumulate by 48 hpi, coinciding with initial spread to the N-compartment. Infections at 10 MOI showed sparse mNG-positive neurons as early as 24 hpi, with limited N-compartment spread that became extensive by 48 and 72 hpi. At 100 MOI, robust mNG expression was evident in both S- and N-compartments from 24 hpi, with fluorescence intensity increasing substantially through 72 hpi. These results demonstrate the clear dose-dependent replication and anterograde spread kinetics of H129-BF in primary neurons.

### H129-BF replication, Flp expression, and transsynaptic spread in the absence and presence of helper AAV *in vivo*** **

**Fig 6:**
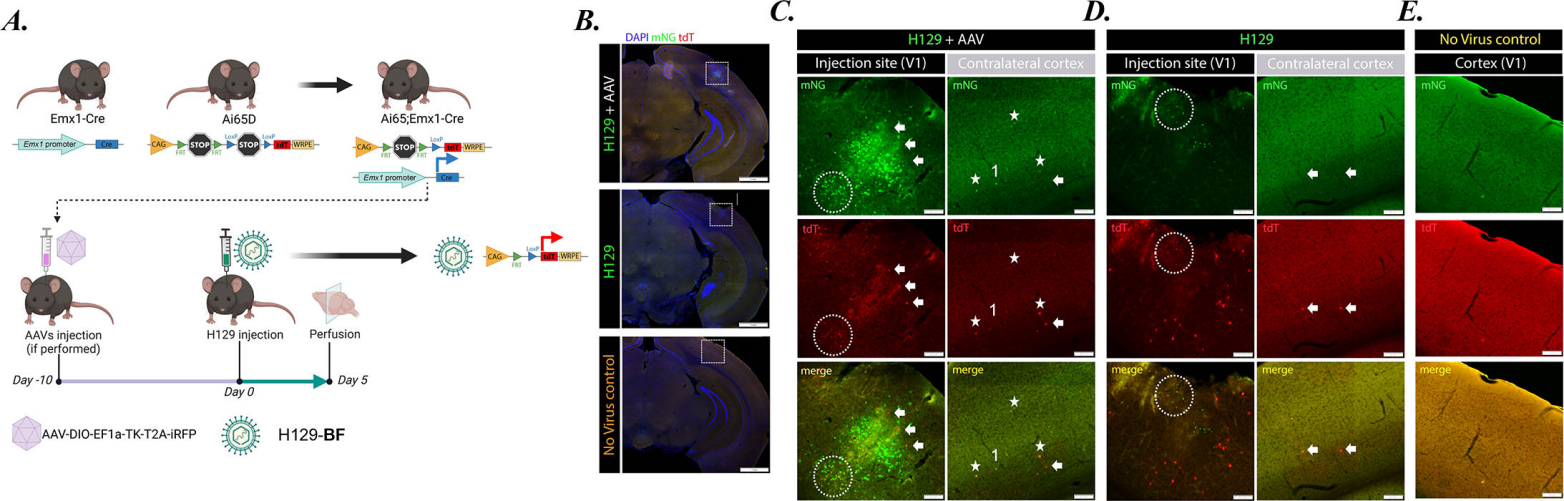
H129-dTK replication and anterograde transsynaptic transport *in vivo.* (**A**) Schematic workflow for the assessment of the H129-dTK transsynaptic transport *in vivo*. Emx1-Cre mice were crossbred to Ai65D mice to generate double transgenic Ai65D; Emx1-Cre mice. Emx1-Cre mice express Cre recombinase in a subpopulation of excitatory neurons under the Emx1 promoter. Expression of tdTomato is dependent on the expression of both Flp and Cre recombinases. Stereotaxic injection of Ai65D; Emx1-Cre at primary visual cortex (V1) with 0.2 µL (1 × 10^5^ pfu) of H129-BF (one group of mice was injected with the AAV-TK; AAV-DIO-TK-EF1a-TK-T2A-iRFP, 10 days prior to H129-BF infection). Five days post-H129 injection, mice were perfused, and brains were fixed and removed for histology (*n* = 2 mice per condition). Created with BioRender.com. (**B**) Injection sites were shown in all three experimental conditions. Injection and contralateral sites are shown in (**C**) H129-BF plus AAV, (**D**) H129-BF alone, and (**E**) no virus injection control. (scale bars are 500 µm). Dashed circles show dual color neurons at the injection site, arrowheads point to single tdT expressing neurons, and stars show dual color neurons at the contralateral site. A single mNG alone expressing neurons at the contralateral site was labeled as 1.

To examine the replication and gene expression dynamics of H129-dTK *in vivo*, we infected dual Cre/Flp reporter mice with H129-BF in the presence or absence of a TK-expressing helper AAV. Emx1-Cre mice express Cre recombinase in a subset of excitatory neurons under control of the Emx1 promoter. The Ai65D reporter line contains a tdTomato (tdT) gene downstream of two transcriptional STOP cassettes: one flanked by FRT sites and the other by LoxP sites. Crossing Emx1-Cre and Ai65D mice generated double transgenic Ai65D; Emx1-Cre dual reporter mice (Fig. 6A). In these mice, tdT expression requires both Flp- and Cre-mediated recombination, with Cre expressed in a defined subset of excitatory neurons.

We stereotaxically injected H129-BF into the primary visual cortex (V1) of the dual reporter mice. A separate group received an AAV helper virus TK and iRFP under Cre control (AAV-DIO-TK-EF1a-TK-T2A-iRFP) 10 days prior to H129-BF infection. Five days after H129-BF injection, mice were perfused, and brains were fixed and processed for histological analysis. Comparison of the injection sites (Fig. 6C and D, first columns) revealed that H129-BF replication and spread, indicated by the viral mNG signal, were markedly enhanced when TK was provided in *trans* by the helper AAV. However, robust viral replication at these sites resulted in local tissue damage (Fig. 6B, top panel).

At the injection sites, we identified three distinct neuronal populations (Fig. 6C): (i) a small subset expressing both Cre and H129-BF-derived Flp recombinase, showing tdT and mNG co-expression (dashed circles); (ii) a few bright tdT-positive neurons lacking mNG signal (arrows); and (iii) numerous mNG-positive neurons with weak tdT labeling (majority of the population). These findings suggest that the kinetics of reporter activation differ between the host-encoded tdT (activated by Flp recombinase) and the virally encoded mNG reporter. We hypothesize that early during infection, low levels of viral Flp expression are sufficient to induce robust tdT production, while mNG expression remains near or below detection. During mid-stage replication, both tdT and mNG can be detected concurrently. As viral replication advances, host translation is progressively suppressed, diminishing tdT expression, whereas viral mNG continues to accumulate.

Anterograde transsynaptic spread of H129-BF was detected in the contralateral cortex, marked by neurons expressing both tdT and mNG (Fig. 6C, second column). Notably, in the contralateral site, the number of tdT-positive neurons exceeded that of mNG-positive cells. We identified three populations representing distinct stages of infection: dual tdT/mNG-positive neurons (stars), singly mNG-positive neurons (1), and singly tdT-positive neurons (arrows). Importantly, H129-BF infection in the absence of helper AAV resulted in limited viral replication at the injection site with minimal tissue damage (Fig. 6B, middle panel). We observed a small number of dual tdT/mNG-positive neurons (dashed circles) and a few neurons expressing only mNG, whereas the majority were tdT-positive only (Fig. 6D, first column). Notably, even without helper AAV, H129-BF exhibited anterograde spread to the contralateral cortex, evidenced by tdT expression (Fig. 6D, second column). At this site, mNG fluorescence from the viral genome was barely detectable, consistent with earlier activation of tdT through Cre- and Flp-mediated recombination. In the control animals that did not receive any viral injections (Fig. 6B, last panel; Fig. 6E), no tissue damage or fluorophore expression was detected. These findings indicate that the absence of a detectable virally encoded fluorophore does not necessarily imply a lack of infection or spread; rather, viral gene expression is likely below the detection threshold, yet sufficient to produce Flp recombinase and induce tdT expression.

### Dose-dependent H129-BF replication and transsynaptic spread without helper AAV *in vivo*** **

**Fig 7:**
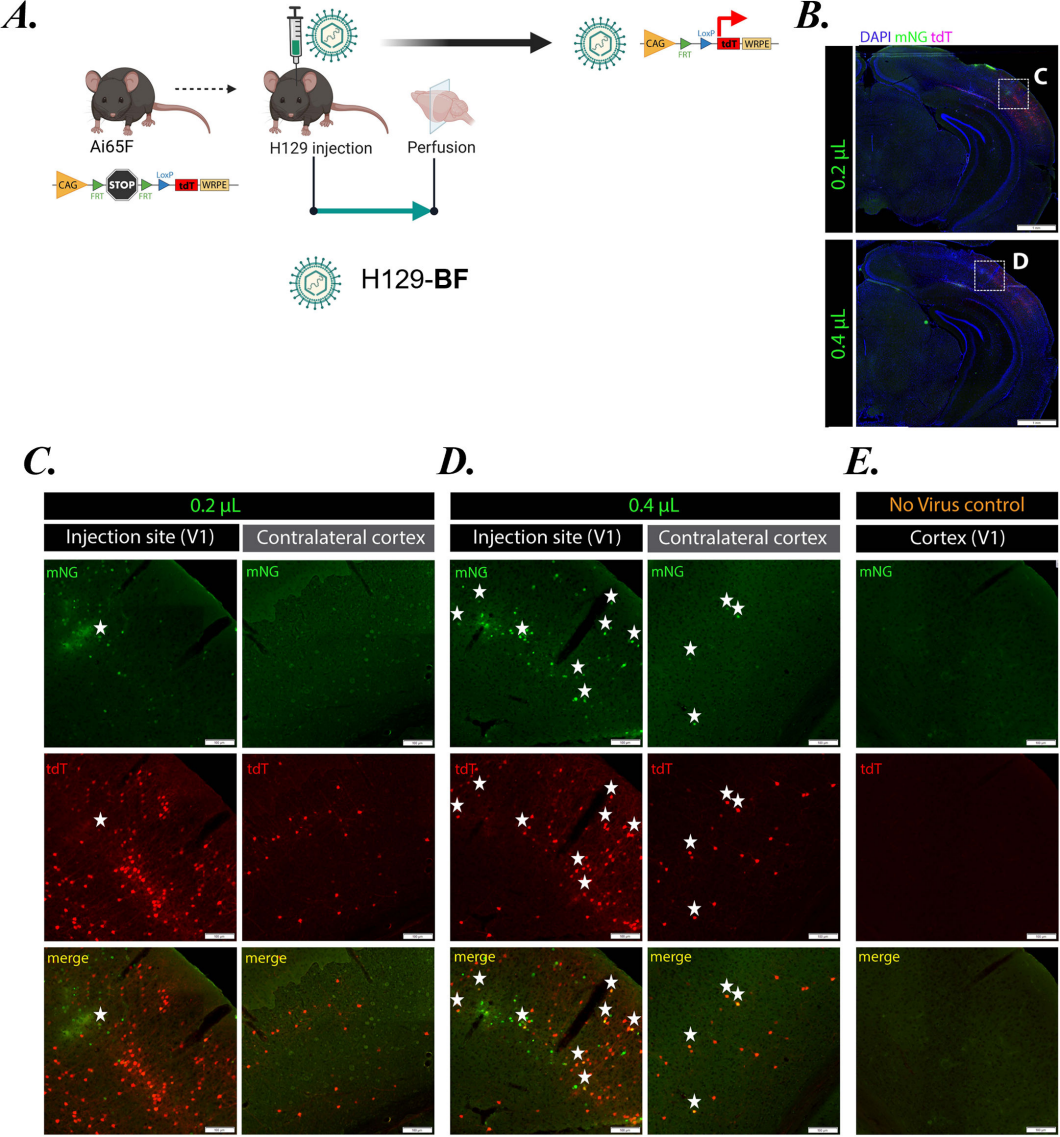
H129-dTK replication and transsynaptic transport increase with dose *in vivo*. (**A**) Schematic workflow: Ai65F mice express tdTomato (tdT) dependent on the expression of Flp recombinase. Stereotaxic injection of Ai65F mice at a primary visual cortex (V1) with H129-BF. Five days post-H129 injection, mice were perfused, and brains were fixed and removed for histology. Created with BioRender.com. (**B**) Injection sites were shown in two different doses of H129-BF infection. H129-BF replication and transsynaptic transport when injected at 1 × 10^5^ pfu (0.2 µL) (**C**) and 2 × 10^5^ pfu (0.4 µL) (**D**) in comparison to no virus infection control (**E**). (scale bars are 500 µm). Cells expressing both tdT and mNG appear yellow in the merge channel and are denoted by stars.

To further examine the replication and spread dynamics of H129-BF in the absence of helper AAV, Ai65F mice were injected in the primary visual cortex (V1) with two doses of H129-BF: 2 × 10⁵ pfu (0.4 µL) and 1 × 10^5^ pfu (0.2 µL) (Fig. 7A). Ai65F mice express tdTomato (tdT) in response to Flp recombinase activity. Mice were perfused 5 days post-injection, and brains were processed for immunohistology. No tissue damage was observed at the injection site without helper AAV, demonstrating the limited replication capacity of H129-ΔTK mutants *in vivo* (Fig. 7B).

Interestingly, mice injected with the lower dose displayed widespread tdT expression at the injection site, with only a few neurons showing single mNG or dual tdT/mNG expression (Fig. 7C, first column; star indicates a dual-color neuron). At this dose, only tdT-positive neurons were detected in the contralateral cortex (Fig. 7C, second column). These results are consistent with our *in vitro* and *in vivo* findings, indicating that H129-BF retains attenuated replication in the absence of TK. Due to this attenuation, viral mNG expression is limited and often below the detection limit, whereas the endogenous tdT reporter shows robust labeling, as only small amounts of viral Flp expression are required to activate the neuronal tdT expression. H129-BF infection, even at a low dose, also labeled anterogradely connected neurons in the contralateral cortex, detectable solely by tdT expression.

Mice injected with the higher dose exhibited a greater number of dual tdT/mNG-positive neurons at the injection site (Fig. 7D, first column; stars). At this dose, we also observed widespread single tdT- or mNG-positive neurons, reflecting different stages of viral infection. The increased replication potential associated with the higher initial viral dose led to more extensive labeling in the contralateral cortex, including a few dual-positive neurons (Fig. 7D, second column; stars) and many tdT-positive neurons.

## DISCUSSION

One of the most widely used polysynaptic tracers in neuroanatomical studies is the HSV-1 patient isolate strain H129, which exhibits a strong preference for anterograde spread (5, 6, 18). The genome of H129 has been fully sequenced (19), but the specific genetic mutations responsible for its anterograde bias remain unclear. Research suggests that defects in retrograde spread may be due to multiple gene mutations that affect viral egress from somato-dendritic regions, promoting preferential axonal transport and egress of progeny virions (5). Over the past two decades, various fluorescently labeled H129 tracers have been developed, many of which replicate constitutively or under conditional control, enabling precise mapping of neuronal circuits in both monosynaptic and polysynaptic tracing studies (20, 23, 31–34).

However, the replication of H129 and its derivatives in the nervous system often leads to neuronal death, a significant drawback in circuit tracing studies (35). The toxicity is primarily due to viral DNA replication and excessive expression of viral proteins. To mitigate this, H129 tracers with TK deletions have been developed (23, 35). Since TK is essential for DNA replication, and its expression is minimal in terminally differentiated neurons, TK deletion slows viral replication in the nervous system (25). While this strategy reduces cytotoxicity, it also limits fluorescent gene expression and often necessitates the complementation of the TK gene via AAV transduction, which introduces additional complexities and challenges, including continued neuronal death (21, 35)

In this present study, we designed and constructed novel H129-dTK recombinant tracers expressing mNeoGreen-P2A-FlpO/Cre from the TK locus, enabling more precise manipulation of neuronal circuits with reduced toxicity. While previous work indicated that TK-null HSV-1 strains have severely limited replication in the nervous system (36, 37), our findings suggest that replication can still occur in certain neuronal subtypes, particularly when infections are initiated at higher doses (i.e., MOIs). We observed dose-dependent replication in cultured SCG and CNs as well as *in vivo* mouse models. This dose-dependent replication capacity aligns with findings showing that TK is not strictly required for viral persistence in the nervous system (38). These results underscore that TK-deleted viruses can still replicate in terminally differentiated neurons if infected with a sufficient virus dose (39). It is essential to carefully consider the infectious virus dose (i.e., virus titer) in the tracing studies, as this parameter influences both the spread efficiency and the survival of the animal. Variability in neuronal permissivity and susceptibility to viral infection further affects the infection dynamics, as certain neuronal populations may be more or less prone to viral infection (40).

The most significant difference in infection dynamics was observed when we infected SCG neurons through their axons. Axonal infections initiated at an MOI of 1 resulted in no detectable viral progeny in the cell body compartment of SCGs, suggesting inefficient retrograde transport and/or establishment of a latent infection under these conditions. However, increasing the MOI to 10 and 100 led to productive infections in half of the samples and all samples, respectively, albeit with lower yields, supporting the hypothesis that replication efficiency is dose-dependent, and axonal entry, retrograde transport, and initiation of the productive cycle are less efficient in the H129-dTK mutants compared to the *wt* H129 virus. It is likely that multiple viral and host factors contribute to this retrograde infection defect, which requires further mechanistic studies.

Despite inefficient retrograde infection, anterograde spread of H129-BF remained relatively unaffected in compartmented SCG neurons when cell bodies were directly infected. When SCG cell body compartments were infected with H129-BF, we detected approximately a 2-log defect in H129-BF progeny production, but the anterograde spread efficiency was not significantly altered, and the N-compartment titers reached wt H129 levels in 3 days.

We further evaluated H129-BF replication and spread in dual-reporter transgenic mice, in the presence or absence of a helper AAV expressing TK under Cre control. In these mice, Cre is expressed in a subset of excitatory neurons, and neuronal tdTomato (tdT) expression requires both Cre- and Flp-mediated recombination. When animals were co-injected with AAV-TK and H129-BF, we observed widespread infection at the injection site, which resulted in marked tissue damage within 5 days. Notably, three distinct fluorophore expression patterns were detected: (i) dual tdT/mNG-positive neurons, as expected, (ii) a small number of tdT-only neurons, and (iii) numerous mNG-only neurons. This observation highlights two key points: first, endogenous and virally encoded reporters exhibit distinct labeling kinetics; and second, neuronal circuit labeling can occur independently of replication-dependent viral gene expression.

Labeling based on virally encoded fluorophores requires robust viral DNA replication and progression into the late phase of infection, during which transcription and translation are upregulated from newly synthesized viral genomes. As host protein synthesis becomes suppressed during this phase, virally encoded fluorophores accumulate to high levels, ultimately leading to neuronal death, corresponding to the (iii) phenotype. In contrast, labeling that depends on virally encoded recombinases (e.g., Flp) to activate an endogenous fluorophore (e.g., tdT) requires only minimal viral gene expression. Small amounts of recombinase are sufficient to permanently activate the reporter, effectively decoupling neuronal labeling from viral replication and minimizing cytotoxicity reflected by the (ii) phenotype, which represents an earlier stage of infection.

In the absence of helper AAVs, H129-dTK mutants exhibited limited replication and anterograde spread in two different reporter mouse models, yet still efficiently labeled anterogradely connected (output) neurons in a dose-dependent manner. Although viral replication was attenuated, recombinase-mediated labeling of connected neurons occurred efficiently without inducing tissue damage or neurotoxicity.

In summary, the replication-dependent stepwise spread of H129-dTK through neuronal circuits presents both opportunities and challenges for circuit-tracing applications. Understanding the kinetics of viral replication and spread, together with the associated neuronal responses, is critical for improving viral tracers. Refinement of these tools, particularly by minimizing neurotoxicity while maintaining labeling efficiency and specificity, will enhance their utility for mapping complex neural networks. Our findings demonstrate that non-complemented H129-dTK recombinants retain limited replication capacity *in vitro* and *in vivo,* yet achieve robust neuronal labeling in appropriate recombinase reporter mouse models with minimal toxicity. Such decoupling of viral replication from neuronal labeling enables more accurate and less invasive long-term circuit analysis. Further optimization of these recombinants will support their development as refined anterograde monosynaptic and polysynaptic transneuronal tracers.

## MATERIALS AND METHODS

### Mammalian cell culture and viruses

Vero cells (African green monkey kidney epithelial cells, CCL-81) were purchased from ATCC and used to grow and titer wt and recombinant H129 viruses. Cre-recombinase-expressing Vero cells were kindly provided by David Leib’s lab at Dartmouth Geisel School of Medicine (27). Vero and Vero-cre cells were cultured in Dulbecco’s Modified Eagle Medium with phenol red (DMEM, Cytiva, Washington, DC, USA) containing 10% fetal bovine serum (FBS, Genessee, Burlington, MA, USA) and 1% 100× penicillin-streptomycin (PS; Cytiva, Washington, DC, USA). HSV-1 strain H129 is a low-passage clinical isolate that was received from Richard Dix (6, 18). For preparing wt or recombinant H129 virus stocks, a master stock was produced in Vero cells infected at a low MOI and harvested when the majority of cells showed a cytopathic effect. The titer of each viral stock was determined as pfu/mL by serial dilution on monolayers of Vero cells under methylcellulose (kindly provided by the Enquist lab, diluted in DMEM; Invitrogen). Viral plaques were stained using methylene blue dye (0.5% methylene blue; Fisher Scientific, 70% methanol; Fisher Scientific) or 1% crystal violet dye (Sigma). AAV1-DIO-EF-1A-TK-T2A-iRFP was made by AAVnergene and provided as a stock at 4.09 × 10^13^ gc/mL.

### Primary neuron cultures

SCGs and CNs were isolated from embryonic day 16–17 Sprague Dawley rat embryos (Charles River Laboratories, Wilmington, MA, USA) as described by Curanovic et al. (29). SCGs were cultured in accordance with the protocol outlined by Salazar et al. (41). Briefly, the SCGs were trypsinized and triturated after isolation and seeded in the (S) compartment of a tri-chamber (0.75–1 per chamber) placed on 35-mm dishes (Fisher Scientific) coated with poly-DL-ornithine (500 μg/mL in pH 8.3 0.1 M borate buffer; Millipore Sigma, Burlington, MA, USA) and mouse laminin (Life Technologies Carlsbad, CA, USA). The SCGs were cultured at 37°C and an atmosphere containing 5% CO2 for 15–25 days to allow for axonal penetration through to the N-compartment from the S-compartment. The neuronal media was prepared with neurobasal medium (Gibco) + 50 × B-27 supplement (Gibco) + 100× Penicillin-Streptomycin-Glutamine (Gibco) + 1,000× murine nerve growth factor (Gibco). SCG cultures were treated with 1 μM Cytosine β-D-arabinofuranoside (Ara-C, Millipore Sigma) to select against any mitotic cells. Two days after Ara-C addition, the medium was removed from the chambers and replaced with fresh neuronal medium. The neuronal medium was changed every 5–7 days. All animal work was done in accordance with the Institutional Animal Care and Use Committee of the University of California, Irvine Research Board under protocol AUP-24-008.

CNs were harvested and cultured as described by Pacifici & Peruzzi, with modifications (42). Briefly, CNs were harvested from day 16–17 rat embryos. Neurons were rinsed with Hank’s Balanced Salt Solution (HBSS) (Gibco). They were then trypsinized, rinsed again with HBSS, triturated, and cultured in 12-well tissue culture dishes at 200,000 cells/well (Laguna Scientific, Laguna, CA, USA) coated with poly-DL-ornithine. They were cultured in neurobasal medium with penicillin-streptomycin-glutamine.

### Virus infection of neuronal cultures

Before directional infection assays (retrograde or anterograde), neuronal methylcellulose (1% methylcellulose diluted in neuronal media) was added to the middle (M) compartment to avoid leakage. For retrograde infection assays, axons in the N- compartments were infected at MOIs of either 1 or 10 via pipetting virus stock directly into the compartment. For anterograde spread assays, neuronal cell bodies in the S-compartments were infected by directly pipetting the virus dilution into neuronal media. The anterograde spread of progeny virus was assayed by seeding Vero cells on axons in the N-compartments and titering the virus yield 24 or 72 hpi. CNs seeded in 12 wells were infected with wt and recombinant H129 viruses at an MOI of 10 via pipetting virus stock directly into the well. Infected cells were harvested 48 hpi, and virus yields were determined via plaque assay. Experiments were carried out with replicate chambers.

### Overlap extension PCR

The overlap extension PCR protocol was adapted from Hilgarth and Lanigan (43). Primers were designed to add a P2A site to the transgene (Table 1). Q5 DNA polymerase (New England Biolabs, Ipswich, MA, USA) was used for all reactions. PCR products were run on 1% agarose gels containing 0.01% ethidium bromide. Gel extraction was performed following the CDS 1 and CDS 2 reactions. All gel extractions described in this study were performed using the Monarch DNA Gel Extraction Kit (New England Biolabs, Ipswich, MA, USA). DNA recovered from gel extraction was used for the overlap PCR to generate the fusion products *mNeonGreenP2ACre* and *mNeonGreenP2AFlpO*. The products of the overlap PCR were then amplified in a final fusion reaction.

**TABLE 1:** Primer sequences used in this study

Primer function	Primer name	Sequence
Flp CDS2 primers	P2fwFlpOprimer	GCCACGAACTTCTCTCTGTTAAAGCAAGCAGGAGACGTGGAAGAAAACCCCGGTCTTATGGCTCCTAAGAAG
	P2revFlpOprimer	CTAGCTAGCTCAGATCCGCCTGTTGATGTAGCTG
Cre CDS2 primers	P2fwCreprimer	GCCACGAACTTCTCTCTGTTAAAGCAAGCAGGAGACGTGGAAGAAAACCCCGGTCTTATGTCCAATTTACTG
	P2revCreprimer	CTAGCTAGCCTAATCGCCATCTTCCAGCAGGCG
CDS1 primers	P1fwmNGprimer	AGGCGCGCCGCCACCATGGTGAGCAAGGGCGAG
	P1revmNGprimer	GTCTCCTGCTTGCTTTAACAGAGAGAAGTTCGTGGCTCCGGATCCCTTGTACAGCTCGTC

### Cloning

H129-dTK-LSL-mCherry plasmid that was kindly provided by Ian Wickersham’s lab at MIT was digested with AscI and NheI (New England Biolabs, Ipswich, MA, USA). Fusion gene products were also digested at the same restriction sites under the same conditions. H129-dTK-LSL was ligated with either *mNeonGreenP2AFlpO* digest product or *mNeonGreenP2ACre* digest product using T4 ligase (NEB).

### Nucleocapsid prep

To isolate H129 genomic DNA, a nucleocapsid DNA preparation was performed as described by Szpara et al. with modifications (44). Briefly, confluent monolayers of Vero cells were infected with wt H129 virus at an MOI of 5 and harvested at 15 hpi. Cell pellets were rinsed and subjected to 1,1,1,2,3,4,4,5,5,5-Decafluoropentane (Sigma) extraction and a glycerol step gradient purification. Viral nucleocapsids were then lysed using SDS and proteinase K. Viral DNA was extracted twice with phenol-chloroform and ethanol precipitation. Viral DNA was collected by a glass hook and resuspended in Tris-EDTA (10 mM Tris, pH 7.6; 1 mM EDTA).

### Virus recombination and plaque purification

Vero and Vero-cre cells were seeded in the absence of antibiotics for transfection experiments. H129-dTK-LSL-mNeonGreenP2AFlpO plasmid DNA was digested with KpnI (New England Biolabs, Ipswich, MA, USA). Co-transfection was performed in a biosafety cabinet using Lipofectamine 2000 (Invitrogen) and Opti-MEM (Gibco). 7.5 µg of HSV-1 H129 genomic DNA and 5.5 µg of digested plasmid DNA were used for co-transfection. H129-dTK-LSL-mNeonGreenP2AFlpO was used to transfect both Vero and Vero-cre cells, while H129-dTK-LSL-mNeonGreenP2ACre was only used to transfect Vero-cre cells. Transfected cells were harvested 72 h post-transfection, pelleted, and frozen at −80℃ for plaque purification. For plaque purification of H129-dTK recombinants, all cells were cultured in DMEM containing 2% FBS, 1% PS, 1% methylcellulose, and 0.02% Thymine 1-β-D-arabinofuranoside (Sigma-Aldrich, St. Louis, MO, USA).

### Western blot

Cell lysates were prepared using RIPA-light buffer including 1 mM dithiothreitol and a protease inhibitor cocktail (Roche). Following a 30-min incubation on ice and sonication, cells were pelleted, and the SN was placed in a new tube with 5× Laemmli buffer and boiled at 95°Cfor 5 min. Immunoblotting was performed by using anti-Beta-actin (1:10,000) (Sigma), anti-HSV-1-TK (1:1,000) (Santa Cruz), and anti-HSV-1 (1:1,000) (Agilent Dako). Secondary mouse or rabbit antibodies (KPL, 1:5,000) were used following primary antibody incubation.

### Microscopy

The Leica (Dmi8) inverted epifluorescence microscope was used with the stage top incubator (Tokai) settings optimized to keep samples at 37°C and an atmosphere containing 5% CO _2_. The images were acquired using 10× or 20× objectives and analyzed using Leica analysis software. Brain slices were imaged using an Olympus VS120 Scanner microscope with a 10× objective.

### Animal experiments and *in vivo* virus injections

All mouse experiments were conducted according to the National Institutes of Health guidelines for animal care and use, and were approved by the University of California, Irvine Institutional Animal Care and Use Committee (IACUC, protocol #: AUP-22-163) and Institutional Biosafety Committee (IBC). Ai65D (JAX strain #: 021875; RRID: IMSR_JAX:021875) and Ai65F (JAX strain #: 032864; RRID: IMSR_JAX:032864) were kept in our colony in the C57BL6/J background (JAX strain #:000664). Food and water were available ad libitum, and the cages contained bedding as standard housing. Animals were kept on a 12-h light/dark cycle (lights on at 6:30 h), and the room temperature was maintained between 21 and 23°C, and humidity was maintained between 40% and 70%.

Mice were first anesthetized under 2-3% isoflurane for about 5 min with a 0.8 L/min oxygen flow rate using an isoflurane tabletop unit (HME109, Highland Medical Equipment, Temecula, CA, USA) in a chamber. After 5 min of isoflurane, the mice’s head fur was shaven before the head was clamped in a stereotaxic frame (Leica Angle Two for mouse, Leica Biosystems Inc., Buffalo Grove, IL, USA) while the body was lying on top of the heating pad (Stoelting Rodent Warmer X1) covered with a clean paper towel. The heat probe is placed under the mouse body. Once in the stereotactic frame, isoflurane flow was resumed, and eye lubricant was applied to each eyeball. The head was disinfected several times with betadine and 70% ethanol before doing a skin incision to expose the skull. A “tips-only” technique was used to keep aseptic purposes. Using a stereotaxic machine with a three-axis micromanipulator and digital atlas (Leica Angle Two for mouse, Leica Biosystems Inc., Buffalo Grove, IL, USA), the targeted injection site was determined using bregma and lambda. A small craniotomy was made above the injection site, exposing the dura. The virus was loaded into a glass pipette (tip diameter, ~20–30 μm) and delivered to the target region using pressure via a picospritzer (General Valve, Hollis, NH). The virus was delivered at a rate of ~0.2μL/5 min for intracranial injections, while a rate of ~2μL/2 min was used for intracranial injections. The glass pipette was kept in place for 10 min after virus delivery. Once the pipette was removed, the incision was closed with staples and tissue adhesive (3 M Vetbond, St. Paul, MN, USA). Carprofen was administered subcutaneously (5 mg/kg). The mice were relocated temporarily to a heated recovery cage until they recovered from anesthesia, then moved back to their home cage.

### Histology

At 5 days post-H129 injections, transcardiac perfusion with heparinized PBS followed by 4% PFA was done in deeply anesthetized mice. Brains were kept in 4% PFA overnight at 4°C for less than 24 h, before being transferred to 30% sucrose PBS solution at 4°C. After brains sunk in the tube, brains were sectioned in 30 µm thick slices (Leica SM2010 R). Brain slices that were mounted were counterstained with DAPI for 1 h at room temperature before being mounted. Mounted slices were imaged with the Olympus VS120 Scanner microscope with a 10× lens.

### Statistical analysis

An unpaired Student’s *t*-test was performed, and data were graphed using GraphPad Prism 10.

## Data Availability

The HSV-1 H129 recombinant viruses generated in this study will be made available upon request through the Viral Core at the Center for Neural Circuit Mapping (CNCM), University of California, Irvine (https://cncm.medschool.uci.edu/viral-core-2/).
